# Association Between Periodontal Status and Oral Health-Related Quality of Life: A Cross-Sectional Study

**DOI:** 10.3390/medicina62071374

**Published:** 2026-07-17

**Authors:** Ivan Ivanov, Anjelika Velkova, Emilia Naseva

**Affiliations:** 1 Department of Periodontology, Faculty of Dental Medicine, Medical University of Sofia, 1431 Sofia, Bulgaria; 2 Department of Social Medicine, Faculty of Public Health “Prof. Tzecomir Vodenitcharov, MD, DSc”, Medical University of Sofia, 1431 Sofia, Bulgaria; a.velkova@foz.mu-sofia.bg; 3 Department of Health Management and Health Economics, Faculty of Public Health “Prof. Tzecomir Vodenitcharov, MD, DSc”, Medical University of Sofia, 1431 Sofia, Bulgaria; e.naseva@foz.mu-sofia.bg

**Keywords:** oral health-related quality of life, periodontitis, OHIP-14, sleep quality, periodontal disease, logistic regression

## Abstract

*Background and Objectives:* Periodontal diseases are highly prevalent worldwide and have been associated with impaired oral health-related quality of life (OHRQoL). However, the strength and direction of this association remain inconsistent, particularly when sociodemographic and behavioural factors are considered. The aim of this study was to evaluate the independent association between periodontal status and OHRQoL in Bulgarian adults attending a university dental clinic, while accounting for sociodemographic factors and sleep quality, which was included as a clinically relevant behavioural determinant of oral health-related quality of life. *Materials and Methods:* This cross-sectional study included 504 adult participants (≥18 years) who underwent comprehensive periodontal examination and completed validated questionnaires. OHRQoL was assessed using the culturally adapted Bulgarian version of the Oral Health Impact Profile (OHIP-14), and sleep quality was measured using the Pittsburgh Sleep Quality Index (PSQI). Due to the skewed distribution of OHIP-14 scores, the outcome was dichotomized at the sample median. Multivariable logistic regression analysis was performed to assess independent associations, adjusting for age, sex, education, financial status, place of residence, and sleep quality. *Results:* Periodontal status, sex, and sleep quality were independently associated with impaired OHRQoL. Females demonstrated lower odds of impaired OHRQoL compared to males (OR = 0.636; 95% CI: 0.428–0.924; *p* = 0.025). Poor sleep quality was associated with increased odds of impaired OHRQoL (OR = 1.554; 95% CI: 1.041–2.321; *p* = 0.031). Periodontitis was significantly associated with impaired OHRQoL (OR = 3.526; 95% CI: 2.073–5.998; *p* < 0.001), reflecting a complex relationship between clinical periodontal status and subjective health perception. *Conclusions:* OHRQoL is influenced by a multifactorial framework integrating periodontal status, behavioural factors, and sociodemographic characteristics. These findings highlight the importance of incorporating patient-reported outcomes into periodontal assessment and support a biopsychosocial approach to oral health research.

## 1. Introduction

Epidemiological studies indicate that more than half of the adult population worldwide is affected by some form of periodontal disease [1]. Compared with earlier reports, these figures suggest a substantial increase and represent a significant public health concern [2]. Their progressive inflammatory nature and cumulative tissue destruction may lead to tooth loss, impaired masticatory function, and increased treatment needs [3]. Beyond their clinical manifestations, periodontal diseases have been associated with systemic inflammation and various chronic conditions, highlighting their broader impact on general health [4].

In addition to their biological and functional consequences, periodontal diseases may substantially affect patients’ daily functioning and psychosocial well-being. Difficulties related to mastication, oral discomfort, impaired aesthetics, halitosis, and tooth mobility may negatively influence self-confidence, social interactions, and emotional health. As a result, the impact of periodontal diseases extends beyond traditional clinical indicators and may significantly alter patients’ subjective perception of oral and general well-being.

Clinical assessment of periodontal status is primarily based on clinical parameters such as probing depth and clinical attachment loss. Although these measures are essential for evaluating disease severity, they do not fully reflect patients’ subjective perceptions or the functional and psychosocial consequences of periodontal conditions in daily life.

In this context, oral health-related quality of life (OHRQoL) has been widely used to assess the extent to which oral health may affect an individual’s daily functioning, psychological well-being, and social interactions. Instruments such as the Oral Health Impact Profile (OHIP-14) allow for standardized assessment of these subjective dimensions and complement the standard clinical examinations [5].

In recent years, increasing interest has been directed toward patient-reported outcome measures in periodontal research and clinical practice. These measures provide important complementary information regarding the subjective burden of disease and treatment outcomes from the patient’s perspective. OHRQoL assessment has therefore become an important component of contemporary periodontal evaluation, particularly in studies aiming to explore the broader functional, emotional, and social impact of periodontal diseases.

Recent evidence suggests that sleep quality may represent an important behavioural determinant of oral health-related quality of life. Sleep disturbances have been associated with impaired oral health perception and poorer quality of life outcomes, while emerging evidence also indicates associations between sleep quality and periodontal status [6,7,8]. Therefore, sleep quality may act as an important factor influencing the relationship between periodontal status and OHRQoL and was included in the present analytical model.

Poor sleep quality has been associated with increased systemic inflammation, altered immune regulation, pain amplification, and impaired psychosocial functioning, all of which may influence both periodontal health and patient-reported quality of life outcomes. Therefore, sleep quality was considered a biologically and clinically relevant covariate in the present study and was included in the multivariable analyses to better characterize the factors associated with oral health-related quality of life.

Although several studies have suggested an association between periodontal diseases and impaired OHRQoL, the available evidence remains inconsistent, particularly when different periodontal diagnoses and disease severities are considered, including periodontal health and disease [9,10,11]. Moreover, epidemiological data regarding the relationship between periodontal status and OHRQoL in the Bulgarian population remain scarce, particularly in studies integrating behavioural and sociodemographic determinants. Based on previous evidence demonstrating poorer oral health-related quality of life among patients with periodontitis, we hypothesized that periodontitis would be associated with impaired OHRQoL. We further hypothesized that sleep quality and sociodemographic factors would independently contribute to OHRQoL variation.

Therefore, the aim of the present study was to investigate the association between periodontal status and oral health-related quality of life and to evaluate whether sleep quality and sociodemographic factors independently contribute to OHRQoL variation in adults attending a university dental clinic in Bulgaria.

## 2. Materials and Methods

### 2.1. Study Design and Population

This cross-sectional observational study was conducted in 2025 at the Faculty of Dental Medicine, Medical University of Sofia, Bulgaria. A total of 504 consecutive adult patients attending the university dental clinic (convenience sample), aged 18 years and older, who attended the clinic during the study period were included in the analysis.

All participants underwent a comprehensive periodontal examination and completed a structured questionnaire including original sociodemographic questions and validated international instruments related to oral health-related quality of life and sleep quality.

Participants were included if they were aged ≥18 years, able to provide informed consent, and capable of independently completing the questionnaire. Individuals with incomplete questionnaires or missing clinical data were excluded from the final analysis.

### 2.2. Questionnaires and OHRQoL Assessment

Oral health-related quality of life was assessed using the Oral Health Impact Profile short form (OHIP-14), one of the most widely used instruments for evaluating subjective oral health burden. The questionnaire assesses functional limitation, physical pain, psychological discomfort, physical disability, psychological disability, social disability, and handicap associated with oral conditions.

The instrument was translated into Bulgarian following a standardized forward-backward translation procedure and reviewed by a qualified bilingual translator. A pilot study involving 80 participants was conducted to evaluate the clarity and comprehensibility of the translated version. Based on pilot testing, minor linguistic modifications were introduced before implementation in the present study.

The questionnaire was self-administered under supervision. Internal consistency of the Bulgarian version demonstrated excellent reliability (Cronbach’s α = 0.935).

### 2.3. Ethical Considerations

The study protocol was approved by the Ethics Committee of the Medical University of Sofia (Protocol No. 17/23.07.2025). The study was conducted in accordance with the ethical principles of the Declaration of Helsinki. All participants provided written informed consent prior to inclusion.

### 2.4. Statistical Methods

Categorical variables are presented as frequencies (*n*) and proportions (%); continuous variables are reported as median and interquartile range (IQR) due to non-normal distribution. Normality was assessed using the Kolmogorov–Smirnov test. Comparisons between two groups were performed using the Mann–Whitney U test for continuous variables and Pearson’s chi-square test for categorical variables. All statistical tests were two-tailed, and statistical significance was set at 0.05. Statistical analyses were performed using IBM SPSS Statistics for Windows, Version 29.0 (IBM Corp., Armonk, NY, USA). Periodontal diagnoses were initially recorded according to the 2017 World Workshop Classification of Periodontal and Peri-Implant Diseases and Conditions. For multivariable analyses, periodontal status was subsequently dichotomized into periodontitis present/absent due to the relatively small number of participants in several individual stage-grade categories and to preserve statistical power. Due to the skewed distribution of OHIP-14 scores, the outcome was dichotomized at the median for logistic regression analysis. Sleep quality was included in the multivariable models based on previous evidence suggesting associations between sleep disturbances, oral health perception, and periodontal disease. In addition, age, sex, education, financial status, and place of residence were included as covariates because previous epidemiological studies have consistently demonstrated their associations with oral health-related quality of life and periodontal health outcomes. The selection of variables was therefore based on biological plausibility and evidence from the existing literature rather than data-driven procedures. Multivariable logistic regression was performed to evaluate independent associations between periodontal status and impaired OHRQoL, adjusting for age, sex, education, financial status, place of residence, and sleep quality. Additionally, multiple linear regression analyses were conducted using continuous OHIP-14 scores to assess the robustness and direction of the observed associations.

Multicollinearity among the independent variables was assessed using variance inflation factors (VIF) and tolerance statistics. No evidence of problematic multicollinearity was observed, as all VIF values were below 2.0 and all tolerance values exceeded 0.50.

### 2.5. Periodontal Examination

Periodontal examination was performed by calibrated dental professionals at the Department of Periodontology. Clinical assessment included evaluation of probing depth, clinical attachment loss, and bleeding on probing. Periodontal diagnosis and classification were established according to the criteria of the 2017 World Workshop on the Classification of Periodontal and Peri-Implant Diseases and Conditions.

Participants were categorized according to periodontal status into periodontally healthy individuals and patients diagnosed with periodontitis.

## 3. Results

### 3.1. Baseline Characteristics

A total of 504 adult participants were included in the final analysis. Baseline demographic and clinical characteristics of the study population are presented in Table 1.

Participants with periodontitis were generally older compared to periodontally healthy individuals. The distribution of periodontal disease also differed according to place of residence and self-reported financial status. In contrast, no statistically significant differences were observed regarding sex or educational level between the study groups.

These findings suggest a non-random distribution of periodontal disease across sociodemographic strata and support the inclusion of these variables in the subsequent multivariable analyses.

Among the 251 participants diagnosed with periodontitis, Stage III disease was the most prevalent form, accounting for 48.2% of all periodontitis cases, followed by Stage IV disease (26.7%). Early-stage disease (Stages I and II) represented a smaller proportion of the affected participants (17.6% combined). Detailed distribution according to periodontal stage is presented in Table 2.

Because Stage III and Stage IV periodontitis accounted for 74.9% of all periodontitis cases, periodontal status was entered into the multivariable regression model as a dichotomous variable (periodontitis present/absent) to preserve statistical power and avoid sparse categories. This approach was chosen because the primary objective of the multivariable analysis was to evaluate the overall association between the presence of periodontitis and OHRQoL rather than differences between individual disease stages.

### 3.2. OHIP-14 Distribution

**Figure 1:**
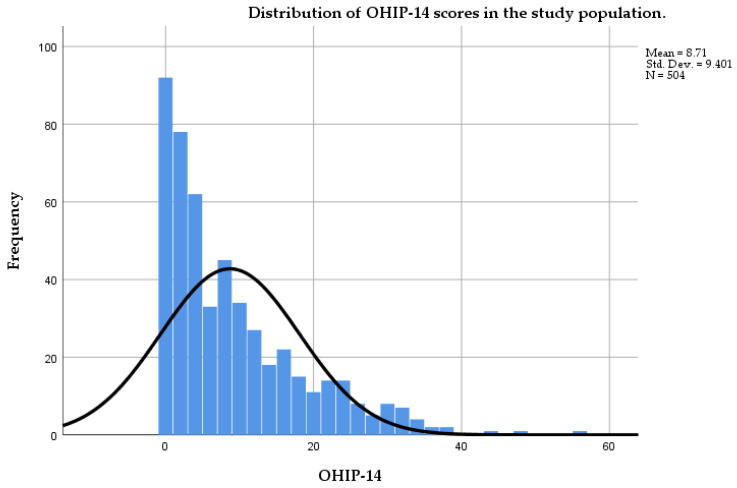
Distribution of OHIP-14 scores among the 504 study participants. The distribution was markedly right-skewed, with a median score of 6 (IQR 1–13), indicating that most participants reported relatively low levels of oral health impact, whereas a smaller proportion experienced substantially impaired oral health-related quality of life.

The median OHIP-14 score was 6 (IQR 1–13), with values ranging from 0 to 56. The distribution was markedly right-skewed (Figure 1), indicating that most participants reported relatively low levels of oral health impact, whereas a smaller proportion demonstrated substantially impaired oral health-related quality of life.

The observed distribution pattern suggests considerable heterogeneity in subjective oral health perception within the study population and supports the use of non-parametric statistical methods.

The sample median OHIP-14 score was 6 points and was used as the cut-off value for dichotomization.

### 3.3. Multivariable Logistic Regression Analysis

Given the non-normal distribution of OHIP-14 scores, the outcome variable was dichotomized at the sample median and entered into a multivariable logistic regression model including sex, age group, education, place of residence, financial status, sleep quality, and periodontal diagnosis.

The overall regression model was statistically significant (χ^2^ = 82.226; df = 15; *p* < 0.001), indicating that the included variables collectively contributed to the prediction of impaired oral health-related quality of life.

After adjustment for age, sex, place of residence, financial status, and sleep quality, periodontitis remained the strongest independent predictor of impaired OHRQoL. Patients with periodontitis had significantly higher odds of impaired OHRQoL compared with periodontally healthy participants (OR = 3.526; 95% CI: 2.073–5.998; *p* < 0.001). Poor sleep quality was also independently associated with impaired OHRQoL (OR = 1.495; 95% CI: 1.010–2.214; *p* = 0.044), whereas female participants demonstrated lower odds of impaired OHRQoL than males (OR = 0.634; 95% CI: 0.431–0.933; *p* = 0.021). Age, place of residence, and financial status were not independently associated with OHRQoL after multivariable adjustment.

Poor sleep quality was associated with significantly increased odds of impaired OHRQoL (OR = 1.495; 95% CI: 1.041–2.321; *p* = 0.031). In addition, female participants demonstrated lower odds of reporting impaired OHRQoL compared with males (OR = 0.634; 95% CI: 0.429–0.947; *p* = 0.026). Financial status demonstrated a trend toward statistical significance (*p* = 0.067), suggesting a possible contribution of socioeconomic conditions to subjective oral health perception.

### 3.4. Additional Linear Regression Analysis

To further evaluate the robustness of the observed associations, an additional multiple linear regression analysis was performed using continuous OHIP-14 scores as the dependent variable (Table 3).

Model statistics: R = 0.429, R^2^ = 0.184, adjusted R^2^ = 0.174, F = 18.641, *p* < 0.001.

Dependent variable: continuous OHIP-14 score. Predictors: age, sex, place of residence, financial status, sleep quality (PSQI > 5), and periodontal status (periodontitis vs. no periodontitis).

The overall model was statistically significant (F = 18.641, *p* < 0.001), with a multiple correlation coefficient of R = 0.429 and an explained variance of R^2^ = 0.184 (adjusted R^2^ = 0.174).

Periodontal status demonstrated the strongest association with OHIP-14 scores (β = 0.321, *p* < 0.001), followed by sleep quality (β = −0.168, *p* < 0.001). Financial status (β = 0.116, *p* = 0.007) and sex (β = 0.109, *p* = 0.008) were also independently associated with OHRQoL. Age and place of residence did not demonstrate statistically significant associations within the adjusted model. These findings were generally consistent with the results of the logistic regression analysis and further support the multifactorial nature of oral health-related quality of life.

## 4. Discussion

The present study investigated the association between periodontal status and oral health-related quality of life in adult patients attending a university dental clinic in Bulgaria. The main findings indicate that sex, sleep quality, and periodontal status were independently associated with impaired OHRQoL after adjustment for relevant sociodemographic factors. These results support the concept that subjective oral health perception is influenced not only by clinical periodontal parameters but also by broader behavioural and psychosocial determinants. The inclusion of sleep quality in the analytical model was motivated by growing evidence linking sleep disturbances with systemic inflammation, periodontal health, and patient-reported quality of life outcomes.

After adjustment for potential confounding factors, periodontitis remained the strongest independent predictor of impaired oral health-related quality of life, increasing the odds of impaired OHRQoL more than threefold. This finding is consistent with previous studies reporting that periodontitis negatively affects oral health-related quality of life and supports the clinical relevance of periodontal status as an independent determinant of patient-reported oral health outcomes.

The association between periodontal disease and reduced quality of life has been consistently reported in the literature. A systematic review demonstrated that patients with periodontitis exhibit significantly higher OHIP-14 scores compared to periodontally healthy controls [9]. Furthermore, the magnitude of impact increases proportionally with disease severity, with severe periodontitis associated with approximately 3.5-fold worse quality of life outcomes. In our study, nearly three-quarters of periodontitis cases in the present study were classified as Stage III or Stage IV disease, indicating a predominance of advanced periodontal destruction within the study sample. This distribution may partly explain the strong association observed between periodontal status and OHRQoL and is consistent with previous reports showing a greater negative impact of advanced periodontitis on patient-reported outcomes. A recent meta-analysis reported that patients in Stage III and IV periodontitis demonstrate significantly impaired OHRQoL, although the effect appears attenuated in the presence of comorbidities [10]. These findings emphasize the independent and clinically relevant role of periodontal disease in shaping patient-reported outcomes. The present findings further support the growing emphasis on patient-centered approaches in periodontal care. Traditional clinical parameters alone may not fully capture the broader functional and psychosocial burden experienced by patients. Therefore, incorporation of OHRQoL measures into routine periodontal assessment may improve individualized treatment planning and facilitate more comprehensive evaluation of treatment outcomes.

The significant sex differences observed in the present study are supported by previous investigations [12,13,14]. Evidence suggests that women often report higher OHIP-14 scores, indicating worse perceived oral health-related quality of life. Slowik et al. [13] demonstrated a modifying role of sex, showing a greater impact of periodontitis on OHRQoL among females. However, other studies have reported no significant differences between sexes in OHIP-14 scores among patients with periodontitis [15]. Discrepancies may be explained by cultural context, disease severity distribution, sampling design, and differences in statistical modeling approaches.

The inclusion of sleep quality in the analytical model was based on growing evidence suggesting that sleep disturbances may influence both oral health perception and periodontal inflammatory burden. Sleep quality emerged as a significant factor associated with oral health-related quality of life. Similar findings have been reported in the literature, where poorer sleep quality correlates with worse OHRQoL outcomes. A pilot study demonstrated a positive correlation between PSQI and OHIP-14 (r = 0.214; *p* < 0.05), indicating that poorer sleep quality is associated with greater oral health impact [6]. Comparable associations have been observed in other populations, with statistically significant correlations between PSQI and OHIP-14 (r = 0.329; *p* < 0.001) [16]. Additionally, previous studies have demonstrated that OHRQoL is independently associated with sleep disturbances (OR = 1.069; 95% CI: 1.043–1.096) [7]. Emerging evidence further supports a biological pathway linking sleep disturbances, systemic inflammation, and periodontal disease, suggesting a plausible sleep, inflammation and OHRQoL axis [8]. Because of the cross-sectional design of the present study, the direction of this association cannot be established. It is equally plausible that impaired oral health, oral discomfort, or periodontal symptoms negatively affect sleep quality, resulting in a potentially bidirectional relationship between sleep and oral health-related quality of life. Therefore, the observed association should be interpreted as correlational rather than causal. Several biological mechanisms may contribute to this relationship. Sleep disturbances have been associated with higher systemic inflammation, altered immune regulation, increased cortisol secretion, and impaired tissue repair capacity, all of which may influence periodontal inflammation and subjective health perception. In addition, poor sleep quality may amplify pain sensitivity, fatigue, and psychological distress, thereby negatively affecting perceived oral health-related quality of life.

Although financial status did not reach conventional statistical significance in the multivariable model (*p* = 0.073), the observed trend suggests a potential modifying role of socioeconomic conditions in shaping OHRQoL perception. Previous studies have demonstrated that lower income is independently associated with higher OHIP-14 scores after adjustment for periodontal status [17]. Similarly, odds ratios ranging from 1.3 to 1.8 have been reported for impaired OHRQoL among individuals with lower socioeconomic status [18]. Socioeconomic disadvantage has also been linked to greater prevalence and severity of periodontal disease, indirectly affecting perceived quality of life [19,20].

From a public health perspective, these findings highlight the importance of considering social and behavioral determinants when developing preventive and therapeutic periodontal strategies. Identification of vulnerable population groups with poorer perceived oral health may support more targeted interventions and patient education programs.

Evidence suggests that periodontal therapy may improve subjective quality of life within three months of treatment [21]. However, patients with advanced periodontal stages may continue to report impaired OHRQoL despite clinical improvement, underscoring the importance of early diagnosis and intervention.

The cross-sectional design of the present study does not allow causal inference. Longitudinal investigations are needed to evaluate dynamic changes in OHRQoL following periodontal therapy and to clarify temporal relationships between periodontal status and quality of life outcomes.

### 4.1. Strengths

The present study has several strengths. First, it included a relatively large sample of adult participants (*n* = 504), allowing for multivariable modelling and adjustment for relevant sociodemographic and behavioural factors. Second, periodontal status was assessed clinically rather than self-reported, increasing diagnostic reliability. Third, the use of a validated and culturally adapted Bulgarian version of OHIP-14 with excellent internal consistency (Cronbach’s α = 0.935) enhances the credibility of the patient-reported outcomes. Additionally, the inclusion of sleep quality in the analytical model provides a broader biopsychosocial perspective, allowing for exploration of potential interactions between behavioural, clinical, and subjective determinants of oral health-related quality of life. The simultaneous evaluation of periodontal status, sleep quality, and OHRQoL within a single analytical framework may also be considered a strength of the present study.

### 4.2. Limitations

Several limitations should be acknowledged. The cross-sectional design precludes conclusions regarding causality or temporal relationships between periodontal status and OHRQoL. Reverse associations or bidirectional influences cannot be excluded. Second, dichotomization of OHIP-14 at the sample median, although methodologically justified due to non-normal distribution, may have resulted in some loss of information and statistical power. Third, the study population consisted of patients attending a university dental clinic, which may limit generalizability to the broader community. Therefore, the study sample should be considered a convenience sample rather than a population-based sample and may not be representative of the Bulgarian adult population. Individuals seeking dental care may differ systematically from the general population in terms of disease awareness, symptom perception, or health-seeking behavior. Finally, unmeasured psychosocial variables, such as perceived stress, coping mechanisms, and pain sensitivity, may contribute to variability in OHRQoL and were not included in the present model. Additionally, self-reported questionnaire data may be influenced by reporting or recall bias, which should be considered when interpreting the findings.

## 5. Conclusions

The present study demonstrates that periodontal status, sleep quality and sex are independently associated with oral health-related quality of life among Bulgarian adults attending a university dental clinic. The findings highlight the multifactorial nature of OHRQoL and suggest that subjective oral health perception reflects not only clinical periodontal parameters but also broader behavioural and psychosocial influences. These results underscore the importance of integrating patient-reported outcomes into periodontal assessment and support a biopsychosocial approach in both clinical practice and future research. Longitudinal studies are warranted to clarify temporal relationships and to further explore the dynamic interplay between periodontal health and disease, sleep quality, and quality of life outcomes.

## Figures and Tables

**Table 1:** Demographic characteristics of the studied population.

		Entire Sample *n* = 504			Patients Without Periodontitis *n* = 253			Patients with Periodontitis *n* = 251			*p*
		Median/*n*	IQR/%		Median/*n*	IQR/%		Median/*n*	IQR/%		
Age, years (median, IQR)		40	25	53	25	22	35	52	45	59	<0.001 *
Age	18–34	195	38.7%		184	72.7%		11	4.4%		<0.001 **
	35–49	146	29.0%		55	21.7%		91	36.3%		
	50–64	118	23.4%		8	3.2%		110	43.8%		
	65+	45	8.9%		6	2.4%		39	15.5%		
Sex	Man	228	45.2%		104	41.1%		124	49.4%		0.061 **
	Woman	276	54.8%		149	58.9%		127	50.6%		
Residence	Sofia	382	75.8%		205	81.0%		177	70.5%		0.010 **
	District center	77	15.3%		26	10.3%		51	20.3%		
	Small town	30	6.0%		19	7.5%		11	4.4%		
	Village	15	3.0%		3	1.2%		12	4.8%		
Income	High	71	14.1%		58	22.9%		13	5.2%		<0.001 **
	Medium	357	70.8%		177	70.0%		180	71.7%		
	Low	56	11.1%		11	4.3%		45	17.9%		
	Variable	20	4.0%		7	2.8%		13	5.2%		
Education (college)	Master’s degree	185	36.7%		97	38.3%		88	35.1%		0.055 **
	Bachelor’s degree	66	13.1%		27	10.7%		39	15.5%		
	College	25	5.0%		9	3.6%		16	6.4%		
	Specialized secondary education	85	16.9%		17	6.7%		68	27.1%		
	Secondary education	132	26.2%		101	39.9%		31	12.4%		
	Primary education or lower	11	2.2%		2	0.8%		9	3.6%		

* Mann–Whitney U test; ** Pearson’s chi-square test.

**Table 2:** Distribution of patients with periodontitis according to disease stage (2017 World Workshop classification).

Stage	*n*	% of Periodontitis Cases
Stage I	24	9.6
Stage II	20	8
Stage III	121	48.2
Stage IV	67	26.7
Stabilized periodontitis	19	7.6
Total	251	100%

**Table 3:** Multiple linear regression model for continuous OHIP-14 scores.

Predictor	B	SE	Standardized β	*p*
Age	0.326	0.531	0.034	0.540
Sex	2.060	0.769	0.109	0.008
Place of residence	0.529	0.528	0.041	0.317
Financial status	1.705	0.627	0.116	0.007
Poor sleep quality (PSQI > 5)	−3.206	0.783	−0.168	<0.001
Periodontitis (present vs. absent)	6.027	1.075	0.321	<0.001

## Data Availability

The data supporting the findings of this study are available from the corresponding author upon reasonable request. The data are not publicly available due to ethical and privacy restrictions.

## Abbreviations

The following abbreviations are used in this manuscript:

OHRQoL	Oral health-related quality of life
OHIP-14	OHIP-14 Oral Health Impact Profile-14
PSQI	Pittsburgh Sleep Quality Index
IQR	Interquartile range
CI	Confidence interval
OR	Odds ratio
